# Draft Genome Sequence of *Microbacterium* sp. TNHR37B Isolated from a Heated Aquifer Bore Well of the Great Artesian Basin, Australia

**DOI:** 10.1128/genomeA.00251-17

**Published:** 2017-04-27

**Authors:** Joseph Adelskov, Bharat K. C. Patel

**Affiliations:** a School of Natural Sciences, Griffith University, Brisbane, Queensland, Australia; b School of Earth, Environment and Biological Sciences, Queensland University of Technology, Brisbane, Queensland, Australia

## Abstract

*Microbacterium* sp. strain TNHR37B was isolated from a geothermal bore well sample (50°C) collected from a region of coal seam gas extraction activities. The 3.5-Mb genome with a G+C content of 69.9% contained unique genes, and a low similarity value for average nucleotide identity using BLAST was observed with the available 73 *Microbacterium* sp. genomes.

## GENOME ANNOUNCEMENT

*Microbacterium* sp. strain TNHR37B was isolated on media D (1) enriched with natural gas from a sample collected from a high-temperature bore well (50°C) located in Surat basin near the town of Bundi, Queensland, Australia. Routine cultivation was in aerobic TYEG medium, which contained mineral salts (2) and 0.2% each of tryptone, yeast extract, and glucose. BLAST analysis of the 16S rRNA gene sequence of strain TNHR37B (815 bp) revealed that it was closely related to *Microbacterium lacticum* strain DSM 20427 (98% similarity). The genus *Microbacterium*, originally proposed by Orla-Jensen (3) is a member of the family *Microbacteriaceae*, phylum *Actinobacteria*, and currently comprises 96 species (http://www.bacterio.net). Here, we report the draft genome sequence of *Microbacterium* sp. strain TNHR37B.

TNHR37B was grown to late log phase in aerobic TYEG medium, and high-molecular-weight DNA was extracted using a modification of Marmur’s method (4). A TruSeq library, constructed from the DNA and sequenced on an Illumina MiSeq platform at the Australian Genome Research Facility (AGRF), produced 1,245,912 paired-end reads of 250-bp length but was reduced to 1,201,672 reads after quality filtering (total 60,083,600 bp). *De novo* genome assembly using SPAdes version 3.5.0 (5) produced a draft assembly consisting of four large contigs (*N*_50_ = 1,357,620 bp; mean coverage of 159×) with a genome size of 3,518,558 bp and a G+C content of 69.9%. Annotation of the draft genome sequence using Prokka version 1.12b (6) identified 3,276 open reading frames consisting of 54 RNA genes and 3,222 protein-coding sequences.

Comparative analysis of the genome sequence of *Microbacterium* sp. strain TNHR37B using average nucleotide identity (100-bp fragment, BLAST method; ANIb) revealed that the *Microbacterium* sp. AO20al1 genome (ANIb identity of 92.19%) was the most similar and that the additional 73 *Microbacterium* sp. genomes that were available in GenBank at the time of analysis shared a much lower ANIb identity (between 83.55 and 86%). These ANIb values are well below the proposed bacterial species demarcation threshold of 95 to 96% and correspond to a 16S rRNA gene similarity value of 98.65% (7). The RAST annotation pipeline (8) revealed that *Microbacterium* sp. TNHR37B contained genes related to one-carbon metabolism, fermentation, metabolism of aromatic compounds, osmotic stress, heat shock, and detoxification. as well as genes involved in the metabolism and utilization of mannose, fructose, l-rhamnose, l-fucose, xylose, l-arabinose, d-ribose, mannitol, glycogen, glycerol, lactate, and chitin. Further comparative analysis of the draft genome with the four closest *Microbacterium* sp. genomes, including strain AO20a11, identified 278 genes unique to strain TNHR37B. These unique genes were annotated as metallo-beta-lactamase, alpha-galactosidase (three genes), putative aminoglycoside nucleotidyltransferase, phosphoesterase, chromate transport protein, o-methyltransferase, predicted glycoside hydrolase, aminoglycoside phosphotransferase, coenzyme F420-dependent oxidoreductase, UDP-glucose dehydrogenase, FAD-dependent oxidoreductase, cellobiose phosphotransferase, and putative integral and ABC membrane transporter proteins. The uniqueness of these genes to the *Microbacterium* genus was confirmed by PSI-BLAST against the NCBI NR database.

### Accession number(s).

This whole-genome shotgun project was deposited in DDBJ/ENA/GenBank under the accession number LQGV00000000. The version described in this paper is the first version, LQGV01000000.
